# To Give or Not to Give? Prescribing Antibiotics to the Tonsillectomy Patients in a Tertiary Care Setting

**DOI:** 10.7759/cureus.16405

**Published:** 2021-07-15

**Authors:** Montasir Junaid, Nadeem W Malik, Yasser Abdelsalam Soliman Galbt, Sadaf Qadeer Ahmed, Hareem U Khan, Abdullah Mohammad Alskaini, Musleh Hussain Mubarki, Ali Saad Alshahrani

**Affiliations:** 1 Otolaryngology - Head and Neck Surgery, Armed Forces Hospital - Southern Region, Khamis Mushait, SAU; 2 Otolaryngology - Head and Neck Surgery, Sir Syed College of Medical Sciences, Karachi, PAK; 3 Otolaryngology - Head and Neck Surgery, Stanford University, Palo Alto, USA

**Keywords:** antibiotics, pediatric population, complications, tonsils, tonsillectomy, post-tonsillectomy bleeding

## Abstract

Introduction

Adeno-tonsillectomy is one of the most common procedures performed worldwide in pediatric age group. Antibiotics use after tonsillectomy is like any other surgical procedure; and it is thought that the antibiotic use may help to reduce post-operative morbidity. Giving antibiotics in tonsillectomy patients is a common practice for decades but recently there has been a paradigm shift towards not using the antibiotics, especially in the pediatric population.

Methods

A prospective study was done on a cohort of 123 patients and they were divided into two groups on the basis of choice to receive or not to receive antibiotics after tonsillectomy, and these patients were followed in post-operative period to see any differences in the rate of complications.

Results

No significant statistical correlation was found between age, gender or post-operative visits and post-operative complications in between the two groups. Half of the patients received antibiotics; however, the use of antibiotics did not show a significant decrease in post-operative complications.

Conclusion

Regular use of antibiotics in post-tonsillectomy patients should not be advised as the use of antibiotics do not prevent or reduce post-operative complications in tonsillectomy patients.

## Introduction

Tonsillectomy is a well-known procedure; defined by the complete removal of tonsil and its capsule from the peritonsillar space [1,2]. This procedure is often performed in day surgery or same-day admission (with or without adenoidectomy). In the US alone, 289,000 tonsillectomies are performed annually in children below the age of 15 years (American Association guidelines) [2]. Like any surgical procedure, tonsillectomy also has associated intra-operative and post-operative complications [3,4]. Intra-operative anesthesia and surgeon-related issues such as trauma to teeth, difficult intubation, laryngospasm, injury to adjacent anatomical structures (lips, tongue, teeth, pharyngeal wall, or carotid artery) have all been reported. After surgery, one of the dreaded complications for a surgeon is postoperative bleeding [5] which usually leads to re-admission and in severe cases re-visiting to the operating room for control of bleed under general anesthesia. This post-operative bleed can be primary (<24 hours) or secondary (>24hours up to 10-12 days). The incidence rate of both is up to 2.2% to 3%, respectively [3]. Other less severe complications in the first two weeks after operation include pain at the operative site, nausea, vomiting, odynophagia, dysphagia dehydration, referred otalgia, and halitosis while later post-obstructive pulmonary edema, velopharyngeal insufficiency, and nasopharyngeal stenosis all have been also reported [3,5-7].

A common understanding was that open tonsillectomy wound or exposed tonsillar bed/fossa serves as a nidus for bacterial (streptococcus pyogene, haemophilus influenza, staphylococcus aureus) growth in oral cavity and may lead to exaggerated inflammatory response leading to exacerbation of post-operative morbidity in these patients and hence the regular use of post-operative antibiotics was warranted [8-10]. First randomized control trial by Talien in 1986, showed less post-operative morbidity in patients who received antibiotics after tonsillectomy when compared to placebo receiving group [11]. Similar results were published thereafter [9,10]. While some studies failed to show any benefit of post-tonsillectomy antibiotics use and advised against it and this helped in avoiding, adverse drug reactions, anti-microbial resistance, preventing unnecessary financial constraints to patients [4,12-13].

Currently, the American Society Guidelines [2] advice against the use of antibiotics in post-tonsillectomy patients. Our hospital policy still remains the one to give antibiotics in post-operative patients in general. In this study, we aim to identify the necessity of giving antibiotics after tonsillectomy surgery in our population and to look for any differences after prescribing or not prescribing antibiotics. This study will help in making decisions by surgeons whether to give or not to give antibiotics to their patients in the future in our population.

## Materials and methods

The study was conducted at the Department of Otolaryngology - Head and Neck Surgery of Armed Forces Hospital Southern Region from December 2018 to December 2019 after being registered and taking ethical approval from the hospital ethical review committee and signed consent of participants who volunteered in the study or their guardians (in case of a minor).

It was a prospective comparative analysis on a cohort of 123 patients divided into two groups. First, Group (A) received post-operative antibiotics and other Group (B) did not receive the antibiotics. Patients (or guardians) were given the option to choose if they wanted to receive the antibiotics post-operatively or not after detailed discussion and explanation with the primary surgeon, thus accordingly patients were put in the two groups. These two groups were compared for the occurrence of post-tonsillectomy complications. Choice of antibiotics was oral co-amoxiclav for seven days (dose according to weight) or oral cefuroxime (dose according to weight) in patients who had known penicillin allergy. All the patients received the same take-home analgesic i.e., oral paracetamol (dose according to weight).

The sample size was calculated to be 59 patients in each group with a 95% confidence interval (total of 118). All the tonsillectomies included in the study were performed under general anesthesia by two senior surgeons (principal investigators) using the same technique (monopolar diathermy for dissection and bipolar diathermy for hemostasis), admitted in day surgery/ambulatory surgery, while all the patients received the same weight-adjusted doses of anesthetic agents and steroids during intubation and extubation by a senior anesthetist. Patients (or guardians) were given the option to choose if they wanted to receive the antibiotics post-operatively or not.

All the patients admitted in ambulatory surgical daycare for tonsillectomy only were included in the study who had a history of recurrent sore throat with fever (recurrent tonsillitis) and the patient or legal guardian understood and willingly participated in the study after signing informed consent. While the exclusion criteria were the patients with indications of tonsillectomy other than recurrent tonsillitis or booked for adenoidectomy along with tonsil surgery or patients who initially gave consent but later withdrew it, patients who were admitted post-operatively with any intra-operative complications for observation or tonsillectomy done by other than principal investigators or method other than mentioned above or the syndromic patients or with co-morbid, or using any other medications were also excluded.

Post-operative patients were discharged from day surgery and a follow-up was given after four weeks (Hospital policy); while patients were asked to visit the hospital emergency in case of any surgery-related issues and inform the on-call ENT team. Any visit related to a post-operative ER visit for the next 2 weeks was also noted. General demographic details for age, gender, use of antibiotic or not, date of surgery, a post-operative visit to ER, complications, need for admission via ER (emergency) were noted on proforma by reviewing the file after the first follow-up and later transferred for statistical analysis using Stata ver. 16.1 (Stata Corp. 2019. Stata Statistical Software: Release 16. College Station, TX: Stata Corp LLC).

Pearson Chi-square test was used to test the association of antibiotic samples with gender, ER visits, postoperative complications, and IV analgesia in ER. An independent sample t-test was used to compare the mean age and day of posts operative ER visits, proportions of post-operative complications were also compared using t-test, p-values less than 0.05 were considered statistically significant.

## Results

**Figure 1:**
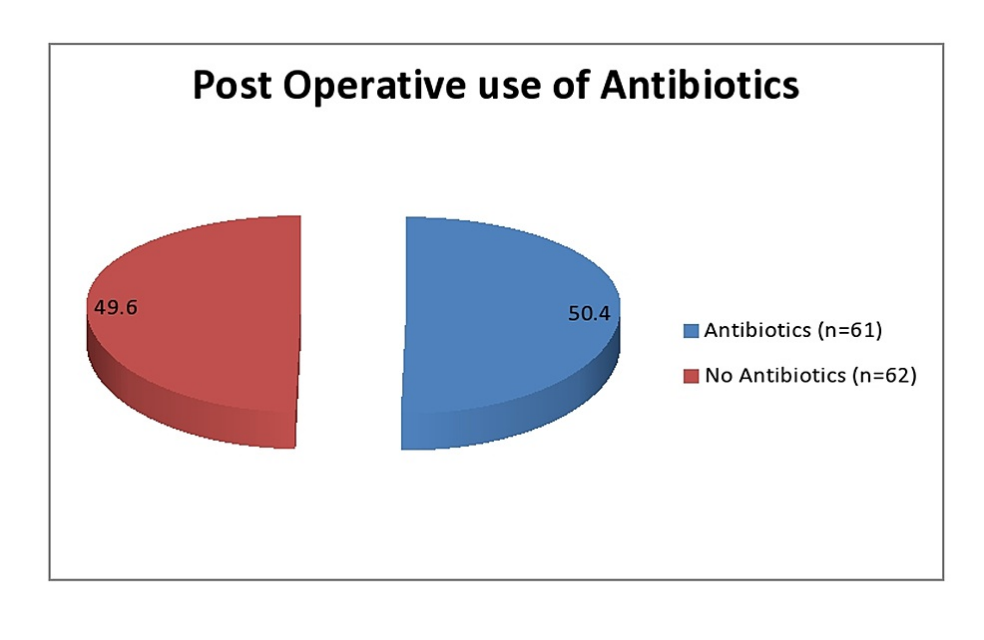
Pie chart.

One hundred twenty-three subjects were included in the study. Sixty-one patients were given antibiotics while 62 patients did not receive any antibiotics (Figure 1).

Table 1 shows the association of the studied parameters after using antibiotics in one group and not giving the antibiotics to the other. There were a total of 60 (48.8%) males while 63 (51.2%) were females. Mean age of patients was 9.5 years ± 7.99. No significant association was found of age, gender and post-operative complications with post-operative use of antibiotics (p > 0.05). The mean days of post-operative ER visit of antibiotic group (5.08 ± 2.74) found significantly higher as compared to non-antibiotic group (2.88 ± 1.35) with p < 0.05. The presenting complaints of patients who visited ER earlier (the group which did not receive antibiotic) were mild to moderate pain which was taken care of. All patients received oral weight-adjusted doses of acetaminophen for postop analgesia with only 20 patients (16%) returning to the ER patients to receive IV analgesics. None of the patients needed re-admission for post op complications.

**Table 1:** Association of studied parameters with the use of post-operative antibiotics. ER: emergency; IV: intravenous. *p-value significant.

Characteristics	Combined, 123 (100)	Antibiotics, 61 (50.4)	No antibiotics, 62 (49.6)	p-value
Male n (%)	60 (48.8)	28 (45.9)	32 (51.5)	0.32
Female, n (%)	63 (51.2)	33 (54.1)	30 (48.4)	
Age in years, (mean ± SD)	9.5 ± 7.99	10.03 ± 8.69	9.14 ±7.25	0.54
ER visits, n (%)	21 (17%)	12 (19.4%)	9 (14.8%)	0.50
Post-op complications, n (%)	21 (17%)	12 (19.4%)	9 (14.8%)	0.49
Day of post-op ER visit (mean ± SD)	4.20 ± 2.50	5.08 ± 2.74	2.88 ± 1.35	0.029*
IV analgesia in ER, n (%)	21 (17%)	12 (19.4%)	9 (14.8%)	0.30

Table 2 shows outcomes of post-operative complications found in the studied samples. Twenty-one (17%) patients had post-operative complications, of which, the most common complication was pain at the operative site which was 57.1% followed by dysphagia (43%), fever (19%) and hemorrhage (14.3%). Results showed a significant difference in the proportion of samples with hemorrhage and otalgia for samples on antibiotics as compared to non-antibiotics.

**Table 2:** Outcomes of post-operative complications. *p-value significant.

Post-operative complications	Combined, 123 (100)	Antibiotics, 61 (50.4)	No antibiotics, 62 (49.6)	p-value
Pain at operative site	12 (57.1%)	6 (37.5%)	6 (46.1%)	0.33
Dysphagia	9 (42.8%)	4 (25%)	5 (38.5%)	0.10
Hemorrhage	3 (14.3%)	3 (18.7%)	0 (0%)	<0.01*
Fever	4 (19.0%)	2 (12.5%)	2 (15.3%)	0.65
Otalgia	1 (4.76%)	1 (6.2%)	0 (0%)	<0.01*

## Discussion

Post-tonsillectomy morbidity prevention remains one of the controversial topics. The common symptoms include dysphagia, odynophagia, dehydration, halitosis, otalgia, or fever [6]. The first 7-10 days are usually difficult for the patients and this has led to interventions such as finding newer techniques of performing tonsillectomy, appropriate analgesia use, ideal antibiotic or not giving it all together, use of steroids, and not limited to home remedies, all of these still fail to provide adequate relief of postoperative morbidity [14]. 

Since a tonsillectomy wound is an open wound inside the oral cavity, bacterial colonization is inevitable leading to secondary inflammatory response; this leads to further post-operative pain and muscle spasm and at times bleeding on already traumatized tonsil bed [8,10]. A safe assumption is antibiotics may help to reduce these symptoms leading to early return to normal activities (diet, school, work, etc.). Though American Association guidelines [2] have advised against regular use of antibiotics in post-tonsillectomy patients, except in cases of post-operative fever. Despite these guidelines; prescribing post-operative antibiotics is still being practiced by many surgeons in America and elsewhere including our institute. Padia et al. analyzed 74 surgeons operating on 15,950 patients concluded that 22% of surgeons still used antibiotics, and no difference in terms of postoperative morbidity (ER visits 7.9%-7.6%, hemorrhage 2.4%-2.5%) was identified in both groups receiving or not receiving antibiotics [15]. In our study return to ER was 14.8% (no antibiotic group) and 19.4% (antibiotic group). Al Jafout et al. In a double-blind study on 270 patients identified that no difference was observed in terms of decreasing post-operative morbidity amongst patients who did not receive any antibiotics pre or post-surgery when compared to patients who receive antibiotics either pre-operatively as IV or post-operative orally as five-day course [13]. In our study, the incidence of postoperative complications in both groups (antibiotic versus no-antibiotic) was the same. While none of them had to be readmitted for secondary hemorrhage but three patients in the antibiotic group had post tonsil bleed which resolved itself with conservative treatment and without in-patient admission. While the ER visits amongst the two groups were also found to be almost similar. Though in our study, we found that the patients who did not receive antibiotics had earlier ER visits (2.88 ± 1.35) compare to the patients who received antibiotics (5.08 ± 2.74). But interestingly, the patients who had earlier visits had presenting complain of mild pain only at the operative site which was dealt with accordingly.

Milder et al. reported data on 5,359 tonsillectomies showed a drop of 86.5% antibiotic use after the publication of American tonsillectomy guidelines (some 14% still adhered to the practice of giving antibiotics) [16]. Upon comparison with before tonsillectomy guidelines (no post-operative antibiotics) statistics, no change in post-operative ER visits, clinic visits, and in-patient admissions was seen but rather a statistically proven rise (1.35%-3.48%, p = 0.009) of surgically treated secondary hemorrhage was observed. In our study, interestingly there was a statistically significant difference between the two groups regarding postoperative hemorrhage and otalgia. Postoperative hemorrhage was found in 3(18.7%) patients who received antibiotics and they were managed conservatively in ER but none of the patients were admitted as in-patients or taken to the operative room for control of bleed. While none of the patients from the group who did not receive antibiotics visited ER with bleeding complain. Though due to the small sample size further studies will be required in the future.

Often the use of antibiotics (especially oral antibiotics may lead to gastric upsets, nausea, vomiting, and allergic reactions in IV antibiotics) have unwarranted side effects [15]. In our study, the patients receiving oral antibiotics did not complain of any drug-related adverse reaction, there is a possibility that most of the patients have been prescribed in the past, multiple antibiotics for their recurrent tonsil infection and hence they tolerated it well. At the same time, repeated use of antibiotics may be harmful to community enlarge with the emergence of the future multidrug drug-resistant organism and increase the financial burden to individual patients and institutes alike [17]. 

Though in literature, the post-operative earlier return to work, fewer post-operative fever episodes, earlier return to eating habits has been attributed to the use of antibiotics [2,18].

## Conclusions

We conclude our study by identifying that there was no significant difference in age, gender and postoperative course in terms of complications, number of ER visits or excessive use of analgesia in both groups. But we found a significant difference in the proportion of post-operative hemorrhage and otalgia among the patients who received antibiotics as compared to the patients who did not receive antibiotics. Though prescribing post-operative antibiotics in tonsillectomy patients is still being practiced, our study clearly shows that post-operative morbidity in patients not receiving antibiotics was no different than the ones receiving it. In light of this study, we can clearly say that antibiotics should not be prescribed unnecessarily to the patients and it is recommended to do further study with a large sample size in the future.
